# The Contribution of Sustained Attention and Response Inhibition to Reading Comprehension Among Japanese Adolescents

**DOI:** 10.3390/children11101245

**Published:** 2024-10-16

**Authors:** Inbar Lucia Trinczer, Yarden Dankner, Shira Frances-Israeli, Yoshi A. Okamoto, Dav Clark, Lilach Shalev

**Affiliations:** 1 Attention Lab, School of Education, Tel-Aviv University, Tel Aviv 67017, Israel; 2 Sagol School of Neuroscience, Tel-Aviv University, Tel Aviv 67017, Israel; 3 SHO-zemi Labs, Shonan Seminar Co., Ltd., Tokyo 150-6222, Japan; okamoto_1990@shozemi.com; 4 Independent Researcher, Towson, MD 21204, USA

**Keywords:** reading comprehension, sustained attention, response inhibition, individual differences, adolescents, Japanese

## Abstract

Background: Previous studies demonstrated the influential role of sustained attention in the reading comprehension of alphabetic writing systems. However, there is limited understanding of how these cognitive functions contribute to reading comprehension in non-alphabetic systems, such as Japanese. This study seeks to explore this gap, focusing on how sustained attention and response inhibition function in a writing system where some of the characters represent meanings rather than sounds, introducing another layer of difficulty in the complex process of reading; Methods: Seventy-five Japanese 9th grade students performed a task to assess sustained attention and response inhibition. The cognitive test was carried out using tablets to enable feasible parallel group administration while maintaining high comparability with ecological classroom settings. Reading comprehension was measured using an exam that the participants took as part of their educational routine; Results: Our results indicate that both sustained attention and response inhibition significantly contributed to the reading comprehension of Japanese 9th grade students; Conclusions: These results replicate and expand previous studies documenting the contribution of sustained attention on the reading comprehension of alphabetic writing systems to a non-alphabetic system. Moreover, our findings unravel another important cognitive factor, namely response inhibition in reading comprehension. We suggest that response inhibition may play a crucial role in reading non-alphabetic writing systems that pose high cognitive demands, such as Japanese.

## 1. Introduction

### 1.1. Background

Reading comprehension is a complex skill fundamental to academic and professional success. The impact of good or poor reading comprehension on learning extends beyond a specific discipline, age group, or culture [1]. The inter-individual differences in reading comprehension proficiency among readers are immense in any given language. Achieving reading comprehension depends on the integration and synchronization of various reading skills, as well as cognitive functions such as word-reading accuracy, word-reading fluency, verbal working memory, and attention, to name a few [2,3,4,5].

Many previous studies based on the Simple View of Reading (SVR) model have focused on individual differences and investigated various language and cognitive abilities that underlie reading comprehension. The SVR model, widely used in reading research, emphasizes the importance of word recognition and language comprehension [6,7,8,9]. According to this model, language-related skills such as phonological awareness and processing, word and sentence decoding speed, vocabulary and syntactic knowledge, and language comprehension are key factors in explaining reading comprehension [10,11,12,13].

Another approach that extends beyond the SVR is the active view of reading, emphasizing the reader’s active engagement in the reading process, and the role of executive functions in reading comprehension [8,9]. Within this framework, previous studies have investigated the input of basic domain-general cognitive factors, such as sustained attention, inhibitory control, and working memory, to reading comprehension [5,10,14]. The current study focuses on investigating the contribution of individual differences in sustained attention and response inhibition to reading comprehension among Japanese adolescents.

Sustained attention, the ability to allocate attention to a prolonged task over time [15], has been associated with reading comprehension in native readers in several languages, such as English, Spanish, and Hebrew [2,5,16]. Stern and Shalev [5] found significant differences in reading comprehension efficiency (among other reading abilities) between adolescent Hebrew readers with either a high, average, or low ability to sustain attention over time. Those with a high ability to sustain attention were better in answering comprehension questions correctly and reading faster than individuals with an average or low ability to sustain attention. In adolescent English readers, Arrington [2] found that good levels of sustained attention facilitate successful reading comprehension by regulating working memory contents while reading. In a study conducted in Mexico with Spanish readers, Silva-Pereyra and colleagues [16] found that elementary school students with poor reading comprehension skills showed a poor ability to sustain attention, compared to those with an adequate grade-level reading ability. However, response inhibition, the ability to withhold a dominant pre-potent response [17], was not found to be consistently predictive of reading comprehension in these languages [5,18,19].

On the other hand, among young Chinese readers, response inhibition has been found to predict reading comprehension in several studies [18,20]. In a cross-cultural study comparing young Chinese and English readers in Canada, response inhibition was found to be predictive of reading comprehension among Chinese readers, but not in English readers [18]. While English, Spanish, and Hebrew use an alphabetic writing system with a relatively small number of characters, the Chinese writing system is logographic and contains thousands of characters [21]. Researchers have previously argued that Chinese readers must often suppress the phonological and semantic activation of similar-looking characters while reading because some Chinese characters may be visually confusing [19,20]. However, it is not clear from the existing data whether response inhibition remains relevant for logographic reading with older children or adults, or whether response inhibition is only relevant during the early stages of learning.

In the current study, we investigated the involvement of two domain-general basic cognitive components in the reading comprehension of another non-alphabetic writing system—Japanese. The Japanese writing system is unique because it combines three scripts: one logographic system—*Kanji* (originating from Chinese characters), and two forms of syllabic scripts: *Kana*—*Hiragana* and *Katakana*. These three scripts are interwoven in Japanese texts, and are used to write different classes of words [14]. Japanese readers must master the three scripts, switching between the orthographic systems while reading texts, and interestingly attain a much faster average reading speed, even in comparison to Chinese [22]. It should be noted that while most previous studies that documented the impact of inhibitory control on Chinese reading comprehension were conducted with preschool and young elementary school children [18,20,23], the participants in the present study were adolescents who are naturally much more experienced with reading and are frequently exposed to academic activities that involve processing complex texts.

Research on gender differences in sustained attention has shown mixed findings. In some studies, males demonstrated faster reaction times and fewer lapses of attention, but they also exhibited more false starts (i.e., responding prior to the target onset) compared to females, suggesting greater impulsivity [24]. Conversely, in tasks such as the Mesulam Continuous Performance Test (M-CPT), females were found to commit fewer errors, indicating better attentional control [25]. However, other studies, such as those using the Sustained Attention to Response Task (SART), found minimal gender differences [26], suggesting variability in results based on the specific task used. Research on gender differences in response inhibition has yielded mixed results as well. Some studies found no significant differences between males and females in inhibitory control [27,28]. Additionally, some studies report a tendency for females to respond faster but with more errors compared to males [29], while others have found that women exhibited slower response inhibition [30].

### 1.2. The Current Study

The aim of the current study was to examine the contribution of two basic domain-general cognitive components, *sustained attention* and *response inhibition*, to reading comprehension among adolescent Japanese readers. Based on previous studies that have demonstrated that sustained attention predicted reading comprehension competency among English, Spanish, and Hebrew readers, and assuming that sustained attention is by definition required in reading texts independent of the writing system type, we hypothesized that good sustained attention would contribute to the proficiency of reading comprehension of Japanese adolescents. Furthermore, since Japanese texts combine syllabic and logographic writing systems, we hypothesized that response inhibition (i.e., the ability to withhold a pre-potent response) would also play a unique role in Japanese adolescent readers’ reading comprehension proficiency. In addition, we sought to explore potential gender differences in these cognitive components, as previous research has shown mixed results.

Additionally, we aimed to collect data in a naturalistic classroom setting to reflect the ecological environment in which students typically engage with reading tasks. The use of tablet-based cognitive tasks facilitated the parallel administration of the cognitive assessment in groups, while maintaining ecological validity similar to real-life classroom conditions [31]. This manuscript not only expands the research on reading comprehension by investigating cognitive factors in the context of Japanese, a non-alphabetic language system, but also employs a tablet-based version of a standard cognitive assessment task, allowing us to examine sustained attention and response inhibition in a context that is more reflective of typical educational settings.

## 2. Materials and Methods

### 2.1. Participants

**Figure 1:**
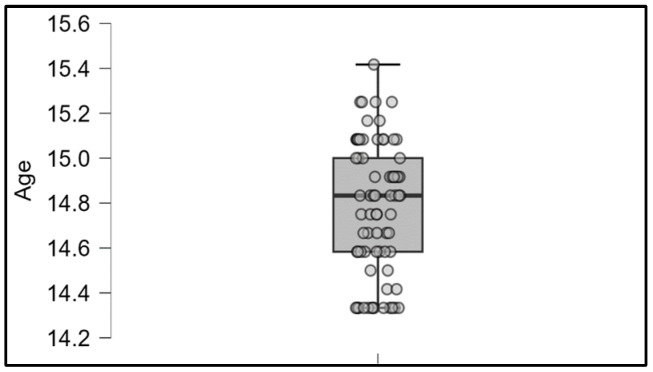
Boxplot of participants’ age in years.

Seventy-five 9th grade Japanese students (thirty-six females) participated in this study. Two participants were discarded from the analysis due to high error rates (see *outliers and data analysis* below), leaving a final sample of seventy-three participants (Mean age = 14.76 years, SD = 0.3). A boxplot of the participants’ age is presented in Figure 1. All participants were students in 2 out of 10 classes in a four-day intensive summer study camp, organized by a private secondary school, in August 2017. The participants were enrolled in a private after-school tuition program specifically designed to prepare students for entry into top-tier high schools. The participants were classified as being at ISCED Level 2, according to the International Standard Classification of Education (ISCED), which corresponds to lower secondary education [32]. As far as we know, none of the participants diagnosed with specific learning disabilities or with attention deficit hyperactivity. No assessment for ADHD symptoms or vision impairments was conducted.

### 2.2. Assessment Tools

**Figure 2:**
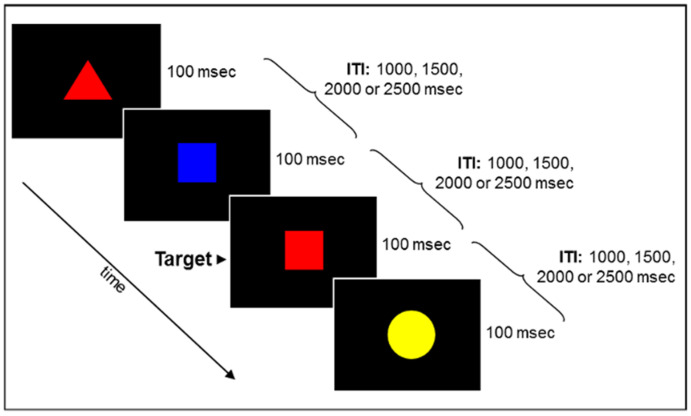
Schematic of the CCPT that was used to assess sustained attention and response inhibition.

*Sustained attention and response inhibition*. The Conjunctive Continuous Performance Test (CCPT) [15] was administered using an OpenSesame version 3.1.7 Android Python 2.7 package on 10.1-inch display Android^TM^ tablets. Each participant performed the CCPT individually on a separate tablet device. The size of each stimulus ranged from 1.2 to 1.8 cm in height, and from 1.2 to 1.9 cm in width. There were 16 possible stimuli resulting from the factorial combinations of a square, circle, triangle, or star appearing in red, blue, green, or yellow (see Figure 2). Each stimulus was presented for 100 milliseconds (ms) and was separated from the next by an interval of 1000, 1500, 2000, or 2500 ms. The various stimulus types and inter-stimulus intervals were randomly intermixed. The target stimulus (i.e., red square) appeared in 30% of the trials, whereas in 17.5% of the trials, a differently-colored square appeared, and in 17.5% of the trials, a red non-square geometric shape appeared. In the remaining 35% of the trials, a non-target shape that shared neither identity nor color with the target appeared. The task consisted of a single block of 320 trials, preceded by 15 practice trials. The task duration was approximately 12 min. Sustained attention and response inhibition were assessed using four measures: means and standard deviations of reaction times of hits (M-RT and SD-RT, respectively), omission errors rate, assessing sustained attention, and commission errors rate, assessing response inhibition. Omission errors refer to the rate of missed targets, and are calculated by dividing the number of omission errors by the total number of targets. Commission errors, on the other hand, refer to the identification of a non-target stimulus as a target, and are calculated by dividing the number of commission errors by the total number of non-targets. Small values of SD-RT and a low omission errors rate reflect a better ability to sustain attention over time. Small values of commission errors reflect a good ability to inhibit responses to non-targets. The split-half reliability coefficient for M-RT is 0.98 and for SD-RT is 0.83, and the test-retest reliability coefficients for M-RT is 0.76 and for SD-RT is 0.62.

*Reading Comprehension.* The Japanese test consisted of *5* problem sets spanning 14 pages and lasted 50 min. It was designed to assess the reading fluency expected of 9th grade students preparing for entrance exams, where proficiency in Kanji and Kana is presumed. All three writing systems are integrated throughout the entire test, including both the questions and the reading materials. The first set was comprised of questions on the understanding of specific particles (auxiliary verbs), and a Haiku (short poem) (20% of the total score). The second set was comprised of questions on classical Japanese reading comprehension of about 700 characters (16% of the total score). The next two problem sets were comprised of long reading comprehension texts in modern Japanese, both of which were over 2 pages long with approximately 3600 to 4000 characters (24% and 30% of the total score, respectively). The final set consisted of questions on graphs and dialogue texts that required students to interpret the content (10% of the total score). The test’s scale ranged from 0 to 100, and the scores of the participants in this study ranged from 42 to 94.

### 2.3. Procedure

The study protocol was approved by an IRB committee. Parents were informed a minimum of two weeks prior to data collection, and students were asked for their assent prior to testing.

*Data collection.* The administration of the CCPT took place during a four-day residential summer tutoring program held at Kuwarubi Hotel, a lakeside resort near Mount Fuji, southwest of Tokyo. This intensive summer program was conducted in August 2017. The CCPT was administered using tablets during the first day of the summer camp, in two different classrooms, to all students simultaneously. Participants were instructed to respond as quickly and as accurately as possible as soon as the target stimulus appeared, and to withhold their responses to all other non-target stimuli. Responses were carried out by touching a dark-grey rectangle, located at the dominant hand side of the screen, using the thumb of their dominant hand. A click sound followed each touch in the dark-grey area.

To assess their reading comprehension, we used the July 2017 Japanese mock exam, which was administered before the summer camp. The Japanese test scores were provided later by the schools’ managers, as part of standard educational practice.

### 2.4. Outliers and Data Analysis

Two participants were excluded from data analyses due to very high rate of omission errors in the CCPT task (>15%). Statistical analyses were conducted using SPSS for Windows version 25 (SPSS Inc., Panasonic, New York, NY, USA). Descriptive statistics (means and standard deviations) were calculated for the CCPT measures (M-RT, SD-RT, omission errors rate, and commission errors rate) and for the Japanese reading comprehension scores. Forward multiple-regression analysis was used to determine if sustained attention and response inhibition (as measured by the CCPT) can significantly predict the reading comprehension of Japanese adolescent students. The Japanese reading comprehension score was used as the dependent variable, and the four CCPT measures were entered as predictors. The significance threshold for the regression analysis was set at *p* < 0.05. Independent sample *t*-tests were used to examine gender differences in reading comprehension and the RT-based measures of sustained attention (M-RT and SD-RT). The *t*-tests were two-tailed, with significance set at *p* < 0.05. Effect sizes were calculated using Cohen’s d. Additionally, for measures that did not meet normality assumptions (i.e., commission errors rate and omission errors rate), the Mann-Whitney U test was applied. As with the *t*-tests, the Mann-Whitney U tests were two-tailed, and significance was set at *p* < 0.05.

## 3. Results

The results of the reading comprehension exam and the various measures from the Conjunctive Continuous Performance Test (CCPT) used to assess sustained attention and response inhibition are presented in Table 1. Descriptive statistics include means, standard deviations, medians, interquartile ranges, skewness, and kurtosis values.

To examine whether sustained attention and/or response inhibition (as measured by the CCPT) significantly predicts Japanese reading comprehension scores, we first conducted a Pearson correlation analysis to assess the relationships between the study variables (see Table 2 for the results). As can be seen from Table 2, reading comprehension significantly correlated with three out of the four CCPT measures: −0.337 with omission errors rate, −0.333 with commission errors rate, and −0.275 with SD-RT. Notably, there was no significant correlation between the omission errors rate and the commission errors rate (r = 0.165, *p* > 0.05). This indicates that these two types of errors reflect distinct aspects of performance: lapses of attention and response inhibition failures, respectively.

Following this, we performed a forward multiple regression analysis, with the Japanese reading comprehension score as the dependent variable and the four CCPT measures as predictors. The regression analysis is summarized in Table 3.

As shown in Table 3, omission errors and commission errors were found to be significant predictors, explaining 19.3% of the variance (R^2^ = 0.193; significance of F change: F_(1,70)_ = 6.865, *p* = 0.011). That is, both sustained attention and response inhibition significantly contribute to reading comprehension among Japanese adolescents. The RT-based measures of sustained attention (M-RT and SD-RT) were excluded in the forward regression model, and were found to be insignificant predictors of reading comprehension (An exploratory Principal Components Analysis (PCA) was conducted on all the study’s variables, including the sub-section scores of the Reading Comprehension test. This additional analysis revealed patterns consistent with those observed in the regression analysis above, which included the overall Reading Comprehension score).

**Figure 3:**
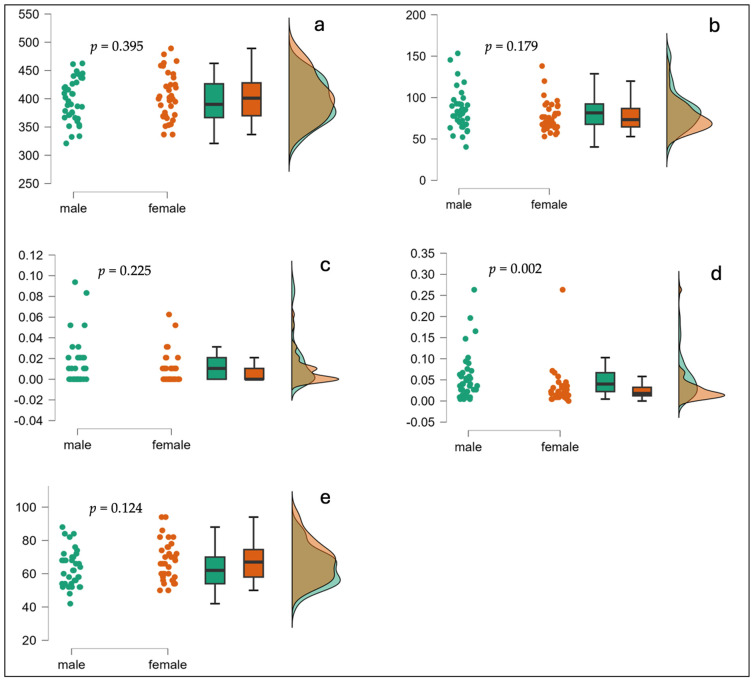
Raincloud plots comparison between genders: (**a**) CCPT—M-RT (ms); (**b**) CCPT—SD-RT (ms); (**c**) CCPT—Omission errors (rate); (**d**) CCPT—Commission errors (rate); (**e**) Reading comprehension score.

Finally, we tested whether there were any gender differences in our sample on reading comprehension, sustained attention, and response inhibition (see Table 4 and Figure 3). There was no age difference between girls and boys (girls: n = 36, mean age = 14.8, SD = 0.30; boys: n = 37, mean age = 14.7, SD = 0.29). We carried out a series of independent sample *t*-tests to compare the performance of boys and girls in the reading comprehension test score, and the RT-based measures of sustained attention (M-RT and SD-RT in the CCPT), which all turned out insignificant (all *p* values > 0.05, two-tailed). The gender differences in commission errors and omission errors were examined by the Mann-Whitney U test. A significant difference was found between boys and girls in the commission errors rate (median = 4% vs. median = 1.8%, respectively; Mann-Whitney U = 392.5, *p* < 0.005, two-tailed) but not in the omission errors rate (Mann-Whitney U = 570, n.s).

## 4. Discussion

In this study, we aimed to replicate previous studies that found sustained attention contributes to reading comprehension in alphabetic writing systems, and expand these findings to a non-alphabetic writing system. Our findings not only confirm the significant role of sustained attention in reading comprehension among adolescent Japanese readers, but also introduce a novel dimension by demonstrating the relevance of response inhibition in this context. Our focus on sustained attention and response inhibition aligns with the active view of the reading model, emphasizing the reader’s active engagement in the reading process, and the crucial role of domain-general cognitive mechanisms in reading comprehension in a non-alphabetic writing system, such as Japanese.

Reading comprehension is a vital proficiency, essential for every learner in literate cultures throughout their entire lifespan [1]. In addition to the various phonological skills, word decoding, and linguistic skills that are necessary to the process of extracting meaning out of written texts [2,3,4,5], several domain-general cognitive factors may impact the efficiency of reading comprehension [5,10,14]. While phonology, decoding, and linguistic knowledge were studied in numerous previous studies, and found to be substantial in different writing systems and languages [10,13], the specific impact of domain-general factors such as sustained attention and/or response inhibition on reading comprehension in different script systems, languages, and cultures was scarcely explored. While the majority of previously aforementioned studies investigated reading comprehension in alphabetic script languages [2,5,16], the Japanese writing system is a unique and highly complex non-alphabetic system that combines three types of scripts simultaneously: a logographic and two syllabic scripts. As a result, Japanese readers must acquire high decoding and linguistic skills to master these different scripts. Moreover, they must switch between orthographic systems when reading texts, and process a large number of characters while refraining from identifying and encoding letter and word errors, which may increase the demand for inhibitory control [14,22].

In this field study, we found that sustained attention was a significant predictor of reading comprehension. This finding replicates previous studies conducted among English, Spanish, and Hebrew readers [2,5,16]. Our results lend further support to the claim that the involvement of sustained attention in reading comprehension is universal and indifferent to the type of orthography in different languages. Interestingly, response inhibition equally and uniquely contributes to the prediction of reading comprehension along with sustained attention in adolescent Japanese readers. In a previous study that used the same neuropsychological task to assess sustained attention and response inhibition (CCPT), response inhibition did not predict reading comprehension among adolescent Hebrew readers [5]. We suggest that response inhibition might be another domain-general cognitive mechanism that plays an important role in reading comprehension in writing systems that pose high cognitive demands, such as Japanese, which requires mastering three scripts including logographic decoding. Further studies are warranted to examine this hypothesis. Interestingly, gender differences emerged specifically in the rate of commission errors, with males showing a significantly higher rate compared to females.

Another contribution of the present study is the age of the participants. While most previous studies that documented the impact of inhibitory control on Chinese reading comprehension were conducted with preschool and young elementary school children [18,20,23], the participants in the present study were adolescents who cope with advanced texts and are required to use reading comprehension skills intensively daily. Our results show that inhibitory control plays an important role in reading comprehension in non-alphabetic writing systems, not only in the early stages of reading acquisition, but also among fluent readers. Notably, the CCPT employed in the present study utilizes simple geometric shapes to assess sustained attention and response inhibition, refraining purposely from using letter-like stimuli. Reading comprehension on the other hand, involves various domain-specific functions, such as letter recognition, word recognition, vocabulary, and many other language skills. Moreover, reading comprehension in Japanese poses high cognitive demands, particularly with the integration of the logographic Kanji system. Hence, this study emphasizes the significant role of basic domain-general cognitive mechanisms, such as sustained attention and response inhibition in reading comprehension in adolescents who use non-alphabetical writing systems. This raises the question of whether cognitive training that addresses attention and inhibitory control [5] can improve reading comprehension.

In the present study, we employed a new tablet version of the CCPT to enable fast and economical group administration in an ecological setting. In all previous studies that have used the CCPT, the task was administered using either desktop or laptop computers [33,34,35,36]. While no direct comparison can be made between the present study and the previous studies, the successful replication of a main finding from the original study on Hebrew readers by Stern and Shalev [5], which used desktop computers and a single rather than group administration, suggests the validity of the sustained attention assessment remained adequate. Despite noisy conditions, the CCPT maintained its psychometric properties in group assessments in the field, and perhaps other similar tasks could be administered feasibly in the future context of large-scale field studies.

Although gender comparisons were not the focus of this study, we tested whether there were differences between boys and girls in reading comprehension, sustained attention, and response inhibition. While no gender differences were obtained in reading comprehension and sustained attention, a significant difference was recorded in response inhibition: boys executed more commission errors than girls, indicating a poorer ability to inhibit pre-potent responses. A similar gender effect was reported in a study that compared young children with and without ADHD using the same task [36]. Yet, this gender difference was not consistent across studies [37,38]. Future research is required to investigate whether gender differences exist in response inhibition, and whether they are linked to cultural biases.

Finally, several limitations should be noted. First, reading comprehension was assessed using a single score taken from a mock exam that participants completed as part of their studies and testing routine. A more detailed assessment of reading comprehension, as well as reading accuracy and fluency, would improve the sensitivity of the reading comprehension assessment. Moreover, future studies may explore the nuances of Kanji-Kana comprehension differences in younger children [39]. Second, since the present study was a field study, the time dedicated to cognitive assessment was limited. Furthermore, the CCPT was administered in the evening of the first day of the summer, after a long and intense day of classes, conditions that may have reduced the reliability of the cognitive measures. Hence, it might be the case that the documented effects are an underestimation of the real effects. Nevertheless, our findings support the approach that basic domain-general cognitive mechanisms contribute to learning efficiency, and that these could be assessed in field studies. Taken together, the present findings point to the important roles of sustained attention and response inhibition in the reading comprehension of adolescent learners. To improve reading comprehension, in light of the present study results, simple adaptations and developments of teaching methods aiming at decreasing sustained attention and/or response inhibition demands should be evaluated. For instance, dividing the studied texts into short units, and embedding various quizzes and short activities, will decrease sustained attention demands and may increase students’ engagement during learning; delaying the response to a question after text-reading by introducing an intermediate stage where students will have to circle the correct answer in the text before responding. Such adjustments could be beneficial for many students.

## Figures and Tables

**Table 1:** Descriptive statistics and normality measures of the reading comprehension score and the Conjunctive Continuous Performance Task (CCPT) measures.

Measure	Mean	SD	Median	IQR	Skewness	Kurtosis
Reading Comprehension Score	65.5	11.37	66	16	0.49	−0.23
CCPT—M-RT (ms)	400	39	400	57.1	0.18	−0.72
CCPT—SD-RT (ms)	80	22	77	25.0	1.27	2.05
CCPT—Omission errors (rate)	0.013	0.021	0.01	0.021	2.23	5.00
CCPT—Commission errors (rate)	0.044	0.052	0.027	0.04	2.76	8.50

Note. CCPT, Conjunctive Continuous Performance Test; SD, Standard Deviation; RT, Reaction Time; ms, millisecond.

**Table 2:** Pearson correlations between each of the variables in the study.

Variable	1.	2.	3.	4.	5.
1. Reading Comprehension Score	—				
2. CCPT—Omission errors rate	−0.337 **	—			
3. CCPT—Commission errors rate	−0.333 **	0.165	—		
4. CCPT—M-RT	−0.118	0.159	−0.278 *	—	
4. CCPT—SD-RT	−0.275 *	0.428 ***	0.109	0.34 **	—

Note. * *p* < 0.05, ** *p* < 0.01, *** *p* < 0.001.

**Table 3:** Regression analysis summary. Dependent variable is the Japanese score.

	B	Std. Error	Beta	t	*p*-Value
Constant	67.927	1.499		45.325	<0.001
Omission errors	−182.481	60.560	−0.337	−3.013	0.004
**R^2^**	0.113				
Constant	70.289	1.699		41.366	<0.001
Omission errors	−158.995	58.890	−0.293	−2.7	0.009
Commission errors	−62.446	23.834	−0.285	−2.62	0.011
**R^2^**	0.193				

**Table 4:** Comparison between boys and girls in reading comprehension, sustained attention, and response inhibition.

Variable	Boys (n = 37)		Girls (n = 36)		t(71)	*p*	Cohen’s d
	Mean	SD	Mean	SD			
Reading Comprehension Score	63.46	10.90	67.56	11.57	1.57	0.124	0.36
CCPT—M-RT (ms)	396	38	404	40	0.856	0.395	0.2
CCPT—SD-RT (ms)	83	23	76	18	1.356	0.179	0.318
**Variable**	**Boys** **(n = 37)**		**Girls** **(n = 36)**		**U(Z)**	***p***	
	**Median**		**Median**				
CCPT—Omission Errors (rate)	0.014		0.007		570 (1.138)	0.255	
CCPT—Commission Errors (rate)	0.04		0.018		392.5 (3.025)	0.002	

Note. CCPT, Conjunctive Continuous Performance Test; SD, Standard Deviation; RT, Reaction Time; ms, millisecond.

## Data Availability

The datasets presented in this study can be found in online repositories. The names of the repository/repositories and accession number(s) can be found below: OSF—https://doi.org/10.17605/OSF.IO/CPZFM. Accessed on 8 August 2022.
